# Bipolar Disorder after Stroke in an Elderly Patient

**DOI:** 10.1155/2014/741934

**Published:** 2014-06-04

**Authors:** Raquel Calvão de Melo, Rui Lopes, José Carlos Alves

**Affiliations:** ^1^Department of Psychiatry, Divino Espírito Santo Hospital, 9500-370 Ponta Delgada, Portugal; ^2^Clinic of Psychiatry and Mental Health, São João Hospital, 4200-319 Porto, Portugal

## Abstract

The onset of bipolar disorder (BD) secondary to a stroke event is a rare clinical entity. Although it may be related to specific regions of the brain, several other factors have been linked to its expression such as subcortical atrophy or chronic vascular burden. While precise locations and cerebral circuits involved in the bipolarity expression after stroke still need to be determined, their investigation represents an opportunity to study brain function and BD etiopathogenesis. We present a BD secondary to multiple subcortical biparietal lacunar infarctions, a lacunar infarction in left putamen and an ischemic lesion at the cerebral trunk evolving the right median portion, in a 65-year-old male patient who experienced manic, hypomanic, and depressive episodes, after 6, 10, and 16 months, respectively, of the cerebrovascular events.

## 1. Introduction

Bipolar disorder (BD) is a severe chronic mood disorder whose onset is commonly associated with early adult life. Among older people in the community, it has been demonstrated in a recent meta-analysis to have a low prevalence of 0.53% [1]; however, it represents approximately 20% of mood disorders [2] and may account for around 8–10% of psychiatric admissions in the elderly [3]. Secondary mania as described by Krauthammer and Klerman [4] has been documented in 17–43% of manic cases in the elderly and has been associated with higher prevalence of cerebral organic disorders [5, 6] such as cerebrovascular disease [7], dementia [8, 9], space occupying lesions, infections, and head injury [10].

Despite the fact that vascular incidents are very high in older people, mania remains a rare clinical entity in this age group [11, 12]. The most relevant imagiological findings in late onset mania are silent cerebral infarctions and also subcortical lesions [11, 13]. These findings, however, need to be considered in the evidence of higher cerebrovascular risk factors in patients with late onset BD that may predict a poor prognosis compared to early onset BD patients [14]. Vascular factors, metabolic abnormalities, and systemic inflammation have been proposed to play an important role in the development of late onset BD [14–16]. But, the question whether a single or multiple lesions are essential for the establishment of secondary BD after stroke is still intriguing investigators, trying to find the exact mechanisms involved. Moreover, whether infarctions lesions could contribute as an allostatic load factor [17] or to accelerate neuroprogression of BD [18] still remains an important field for investigation.

## 2. Case Report

We present the case of a 65-year-old Caucasian man without previous psychiatric history that presented to our outpatient clinic due to elevated mood, reduced need of sleep, pressured speech, thoughts race, elevated energy, and disinhibition with a month of evolution. The worsening of the symptoms led his niece and brother-in-law to make an appointment in our outpatient psychiatry services. According to his family, the patient also started money overspending and abuse of alcoholic drinks and had frequent conflicts with the rest of the family, especially with his son with whom he had an emotional break-up just after the beginning of the symptoms. There was no previous history of manic or depressive symptoms.

The patient is the older of two sons, in a family of middle socioeconomic status. He had a eutocic birth and had normal psychomotor development with no relevant health problems. At 6 years of age he was enrolled in primary school. He was a sociable child and his adolescence had no relevant issues. At 18 years he went to college and took a degree in economics. During that time he met his future wife with whom he would marry after he began working as an economist in a private company at the age of 24, and they had one son. His marriage lasted until 13 years ago when he got divorced and started living alone and after 3 years he also retired from his work.

**Figure 1:**
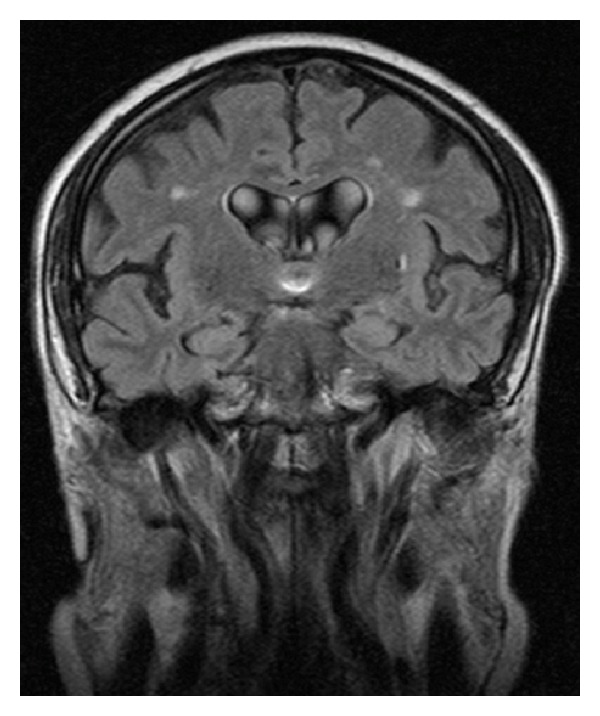
MRI Flair—multiple subcortical biparietal lacunar infarctions.

**Figure 2:**
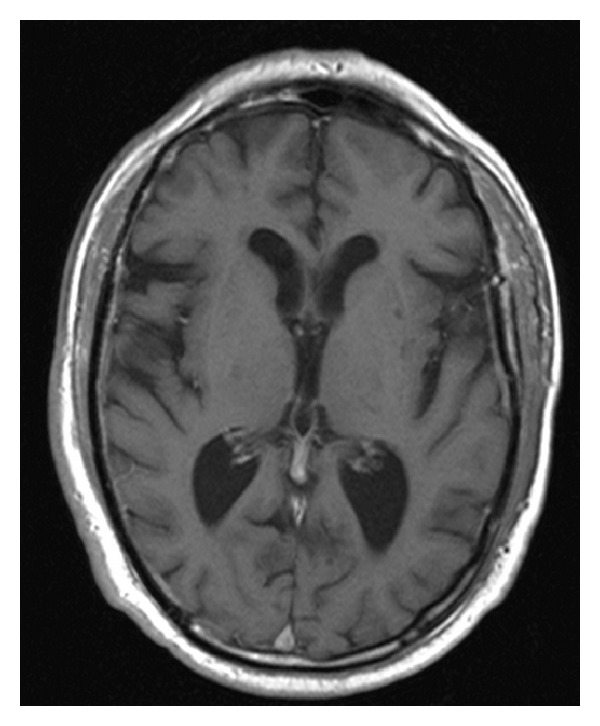
MRI T1—lacunar infarction in left putamen.

**Figure 3:**
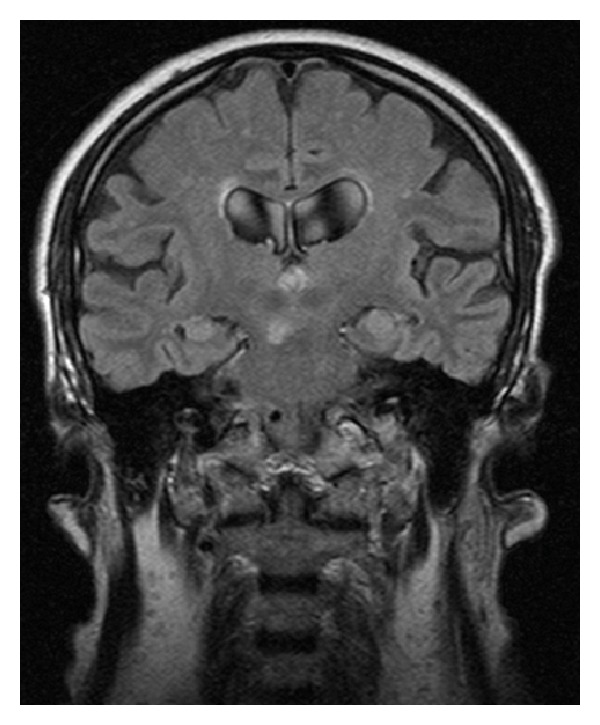
MRI Flair—ischemic lesion at the cerebral trunk evolving the right median portion.

Six months before the beginning of symptoms the patient had a stroke and was admitted at the neurology inpatient service. Computed tomography (CT) scan and brain magnetic resonance imaging (MRI) revealed generalized cerebral atrophy with multiple subcortical biparietal lacunar infarctions (Figure 1), a lacunar infarction in left putamen (Figure 2) and an ischemic lesion at the cerebral trunk evolving the right median portion (Figure 3).

At the neurology inpatient service, he started taking acetylsalicylic acid 100 mg/day and irbesartan/hydrochlorothiazide 150/12.5 mg/day for a diagnosed hypertension. He was already taking simvastatin 20 mg/day for dyslipidaemia. His medical history also revealed that he had been smoking about 30 cigarettes per day for the last years but no illicit drugs consumption.

The patient was resistant to receiving treatment in our inpatient facilities so he was admitted to our day hospital for psychopathological compensation. On the mental examination at admission he presented with good appearance, was alert, cooperative, oriented in time and space, attention procurable but difficult to maintain, and easily irritable, and had elevated mood. He was unable to stay sited and gesticulated more than usual when talking, according to his niece.

The patient had pressure of speech and thoughts race with megalomaniac ideas of grandiosity. There was no hallucinatory activity and patient had no insight or critical appraisal for his clinical situation. Neurological examination revealed a stooped, stiff posture with the head and neck bent forward, dysarthric speech, and discrete hemiparesis in the right hemibody.

We ran a brief test battery to exclude other organic causes, including blood chemistry, thyroid function, folic acid, vitamin B12, C-reactive protein, summary analysis of urine type II, coagulation studies, illicit drugs on urine and alcohol in blood, EEG, chest radiography, HIV, HCV, and HBV viral markers, and syphilis serology, which revealed no significant alterations. He also had a brief screening neuropsychological assessment, scoring 29 points in the Saint Louis University mental status examination (SLUMS), demonstrating apparently normal cognitive functioning (normal score range for high school education patient between 27 and 30) [19, 20].

The patient started pharmacological therapy with diazepam 10 mg/day, olanzapine 10 mg/day, and sodium valproate 1000 mg/day. After a month of inpatient he was asymptomatic, with therapeutic plasmatic levels of sodium valproate (70 mg/L), was discharged, and maintained regular observations in our outpatient clinic.

After 4 months he presented with hypomanic symptoms, so we made adjustments in pharmacotherapy to olanzapine 15 mg/day and diazepam 15 mg/day, maintaining sodium valproate 1000 mg/day that resulted in remission of the symptoms after one week. He remained euthymic in the next 6 months of follow-up till he progressively presented with psychomotor inhibition, terminal insomnia, anhedonia, depressed mood, despair, asthenia, and death thoughts as “a relief from this situation.” We proceeded to another pharmacotherapy adjustment adding bupropion 150 mg once a day and olanzapine and diazepam were slowly reduced each to 5 mg/day. After one month with this therapy he maintained depressive mood; however, the rest of the clinical picture improved and two months later he was asymptomatic.

## 3. Discussion

The “organic hypothesis” of late onset BD implies that patients develop symptoms of mania or hypomania for the first time after having brain lesions [21]. The diagnosis of BD due to another medical condition according to DSM-5 requires a prominent and persistent period of abnormally elevated, expansive, or irritable mood and increased activity or energy predominating in the clinical picture that is attributable to the direct pathophysiological consequence of another medical condition. The manic/hypomanic symptoms usually have acute or subacute onset related to the beginning of the associated medical condition, are not better explained by another mental disorder, and do not occur exclusively during a course of delirium [22].

Our patient presented elevated mood, reduced need of sleep, pressured speech, thoughts race, elevated energy, and disinhibition with a month of evolution and these symptoms appeared 6 months after he suffered a stroke. Before the onset of the stroke our patient was asymptomatic, without a previous psychiatric history. Also, according to DSM-5 diagnostic criteria the disturbance must cause clinically significant distress or impairment in social, occupational, or other important areas of functioning. In our case the patient underwent money overspending and abuse of alcoholic drinks, had frequent conflicts with his family, also needed hospitalization to prevent harm to self or others, and also had psychotic symptoms [22].

The plausibility and probability of causal relationship of the patient's development of BD secondary to the multiple subcortical biparietal lacunar infarctions, a lacunar infarction in left putamen and an ischemic lesion at the cerebral trunk evolving the right median portion, are based on temporal sequence and clinical evidence. Usually, the manic/hypomanic picture due to another medical condition may appear within weeks [22, 23] or a few months [23]. Also, there is absence of evidence to suggest an alternative cause of the mental syndrome, such as a strong family history or precipitating stress [23]. Moreover, patient neuroimaging studies showed generalized cerebral atrophy, which has also been recognized as a vulnerability increasing factor to mania after stroke [24].

Secondary mania and BD after stroke result mostly from focal and solitary lesions, however literature describes correlation between this pathology and lesions with various locations [25]. Although the majority of the studies reporting secondary mania and BD after stroke allocate lesions to the right hemisphere [26–28], a putative effect for laterality on its etiopathogenesis remains under discussion [29–31]. A mechanism implying the activation or release of left hemisphere influence after a right hemisphere contralesion has been proposed [30]. Corroborating this hypothesis, it has been recently demonstrated by comparison of pre- and poststroke single-photon emission computed tomography (SPECT) scans, a left frontotemporal hyperperfusion, and extensive right frontal hypoperfusion in a patient with poststroke mania after right infarction in the territory of the middle cerebral artery [29]. Interestingly, this proposed release of left hemisphere also seems to be present in the manic pole of bipolar syndrome (not postlesion) [32, 33]. We cannot ascertain in our patient that this left hemisphere release phenomenon is the determining factor for the bipolarity expression as the patient presents with lesions on several locations. In fact, it may probably comprise the alternatively proposed hypothesis of the involvement of lesions located on several cortical and subcortical circuits. Starkstein et al. have documented that the majority of patients with right cortical lesions had poststroke mania, oppositely to patients experiencing mania and depression after stroke in right hemisphere subcortical areas [25]. Accordingly, our patient has experienced both mania and depression, as lesions comprised both cortical and subcortical areas.

Moreover, as the localization of lesions was reported on frontal lobe (including orbitofrontal cortex), temporal lobe, basal ganglia (head of caudate nucleus), and thalamus [29, 34, 35], it has been suggested that dysfunction of the frontolimbic circuits could negatively influence mood modulation resulting in the expression of bipolarity through manic symptoms. Due to the involvement of cerebellar-pontine lesions in these circuits [36, 37], the involvement of rostral brainstem dopaminergic nuclei damage and its ascending dopaminergic projections to the frontal-subcortical circuit components in the cases of poststroke mania located in pontomesencephalic area has also been hypothesized [38, 39]. Accordingly, the ischemic lesion at the cerebral trunk evolving the right median portion in our patient could involve also these ascending dopaminergic projections.

BD with late onset is a relatively rare condition and its management must take into account several organic comorbidities. The treatment includes a mood stabilizer (such as sodium valproate or carbamazepine) and a special care should be taken when introducing antidepressants, and also lithium should be used carefully [40]. Due to association of expression of BD in later life with higher cerebrovascular risk, additional research is needed to better understand the interrelationship between the expression of bipolarity and vascular brain lesions and also to determine clinical biomarkers and preventable measures of progression and treatment of BD poststroke. Neuroimaging may play an important role in identifying and staging these risk factors.
